# Simultaneous primary invasive cutaneous aspergillosis in two preterm twins: case report and review of the literature

**DOI:** 10.1186/s12879-017-2646-8

**Published:** 2017-08-02

**Authors:** Floriane Gallais, Julie Denis, Olfa Koobar, Laurence Dillenseger, Dominique Astruc, Raoul Herbrecht, Ermanno Candolfi, Valérie Letscher-Bru, Marcela Sabou

**Affiliations:** 1 Laboratoire de Parasitologie et de Mycologie Médicale, Plateau Technique de Microbiologie ; Hôpitaux Universitaires de Strasbourg. 1 Place de l’Hôpital, F-67000 Strasbourg, France; 2 Université de Strasbourg, Institut de Parasitologie et de Pathologie Tropicale, DIHP EA 7292, Fédération de Médecine Translationnelle, 3 rue Koeberlé, F-67000 Strasbourg, France; 3 Service de Réanimation Néonatale, Hôpital de Hautepierre ; Hôpitaux Universitaires de Strasbourg, Avenue Molière, F-67200 Strasbourg, France; 4 Service d’Oncologie et d’Hématologie, Hôpital de Hautepierre ; Hôpitaux Universitaires de Strasbourg et Université de Strasbourg, Strasbourg, France

**Keywords:** Primary cutaneous aspergillosis, Newborn, *Aspergillus*, *A. fumigatus*, Preterm, Premature, Invasive aspergillosis

## Abstract

**Background:**

Primary invasive cutaneous aspergillosis is a rare fungal infection that occurs mostly in immunocompromised patients. Newborns of very low birth weight present a high risk for this type of infection due to an immaturity of the cutaneous barrier and of the immune system.

**Case presentation:**

We describe here a case of simultaneous invasive cutaneous aspergillosis in two preterm twins. Two male preterm bichorionic biamniotic twins (A & B) were born at a general hospital by spontaneous normal delivery at 24 weeks and 6 days of gestation. They were transferred to our hospital where they receive surfactant, antibiotics and hydrocortisone. Six days later, twin A showed greenish lesions in the umbilical region. The spectrum of antibiotic therapy was broadened and fluconazole was added. The umbilical catheters of the two twins were removed and replaced by epicutaneo-cava venous catheters and the cultures were positive for *Aspergillus fumigatus*. Fluconazole was replaced in both twins by liposomal amphotericin B and the incubators were changed. The serum galactomannan was also positive for both twins. At day 10, yellowish lesions appeared in the abdominal region in twin B. He died on day 18 following complications related to his prematurity. Concerning the twin A, serum galactomannan was negative on day 30; liposomal amphotericin B was stopped 1 week later, with a relay by econazole (cream). His condition improved and on day 66 he was transferred for follow-up at the general hospital where he was born.

**Conclusion:**

The source of contamination by *A. fumigatus* was not identified, but other similar cases from the literature include construction work at or near the hospital, oximeter sensors, latex finger stalls, non-sterile gloves, humidifying chambers of incubators, bedding and adhesive tapes. The skin fragility of preterm newborns is an excellent potential entry point for environmental fungal infections. These cases highlight the importance of suspecting primary cutaneous aspergillosis in extremely low birth weight neonates with rapidly progressive necrotic lesions.

## Background

Primary cutaneous aspergillosis (PCA) is an uncommon fungal infection that occurs mostly in preterm infants and other immunocompromised populations. Neonates of extremely low birth weight are at high risk for this type of infection because of decreased qualitative immune defenses and defects in the skin barrier [1]. We describe here the case of simultaneous abdominal invasive cutaneous aspergillosis in preterm twins, confirmed by fungal culture and high levels of *Aspergillus* galactomannan antigen in the serum.

## Case presentation

Two male preterm bichorionic biamniotic twins were born (day 0) at a general hospital by spontaneous normal delivery at 24 weeks and 6 days of gestation. The 31-year-old mother did not have any particular medical history and did not receive prophylactic antibiotic treatment or antenatal steroids. The pregnancy was obtained by in vitro fertilization and was uneventful until delivery. The second twin (Twin A) was born 3 h after his brother (Twin B). Their birth weights were 614 g (Twin A) and 760 g (Twin B) and the Apgar scores were 5–10 (Twin A) and 3–7 (Twin B) at 1 and 10 min, respectively. Central umbilical vein catheters were placed in both twins after birth. They were immediately intubated and given one dose of surfactant, before being transferred to the Neonatal Intensive Care Unit (NICU) of Hautepierre University Hospital (Strasbourg, France) for further management.

### Twin A: evolution

On admission in NICU, the twin A received one additional dose of surfactant for hyaline membrane disease. An early neonatal infection following a presumed chorioamniotitis was suspected and systemic antibiotic therapy was started with amoxicillin and amikacin, without antifungal prophylaxis. He was treated with low doses of hydrocortisone during the first 10 days of life (0.5 mg/kg, twice a day for 7 days and then once a day for 3 days). This treatment was conducted in application of a new protocol after preliminary results of a study evaluating the efficacy of hydrocortisone in preventing bronchopulmonary dysplasia in very preterm neonates [2].

On day 3 after birth, antibiotics were discontinued because of a normalized C reactive protein (CRP). Six days after birth, twin A presented crusted and greenish cutaneous lesions on the abdomen and at the umbilicus. Intravenous amikacin, cefotaxime, vancomycin and fluconazole (8 mg/kg/d) were initiated. The umbilical catheter was removed in both twins and replaced by a peripheral venous catheter in the axillary vein. On day 8, the culture of this umbilical catheter (sent by the clinical department for bacteriological analysis only) was positive for *Staphylococcus epidermidis* identified by MALDI-TOF mass spectrometry (Brüker Daltonics, Ettlingen, Germany), and for molds, with a count of 20 to 40 fungal colonies on Columbia agar with 5% sheep’s blood (Oxoid, Dardilly, France). Moreover, on the same day, the cultures of swabs from a skin lesion and from the umbilical cord were also positive with more of 40 fungal colonies on ChromID™ *Candida* agar (bioMérieux, Marcy l’Etoile, France), macroscopically and microscopically compatible with *Aspergillus* spp. Fluconazole was therefore replaced on day 8 with liposomal amphotericin B (5 mg/kg/d) with continuation of vancomycin. Incubators were replaced for both twins. *A. fumigatus* was subsequently identified by phenotypic characteristics after culture on 2% malt extract agar (in-house) in all of these samples. Many colonies of a susceptible strain of *Candida guilliermondii* identified by MALDI-TOF mass spectrometry were also found in the culture of a skin lesion. The level of serum *Aspergillus* galactomannan antigen (enzymatic immuno-assay Platelia® *Aspergillus* Ag Bio-Rad, Marnes-la-Coquette, France) was very high on day 9. Indeed, it reached a value >5 (index) with a threshold of positivity of 0.5 (index). *A. fumigatus* was also found in low quantities in urine samples collected on day 9, but the culture of tracheal aspirations as well as blood cultures remained negative.

**Fig. 1:**
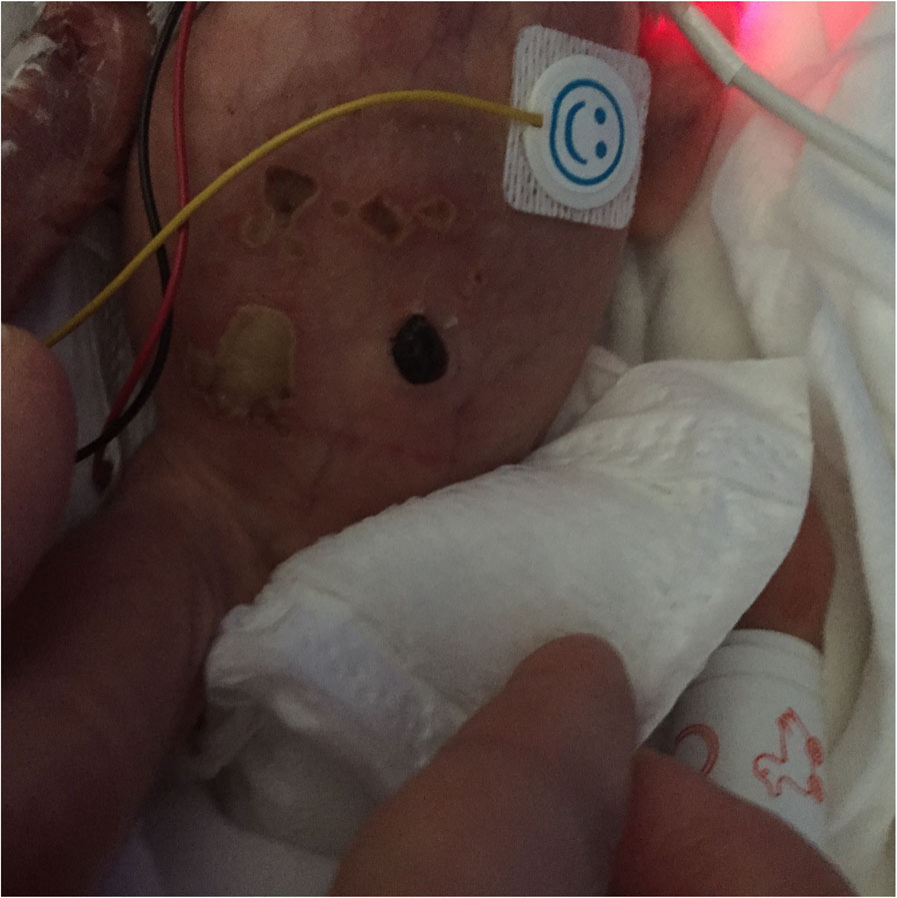
Abdominal lesions of twin A (Day 10)

A second smear of the abdominal cutaneous lesions was collected on day 14. The culture revealed only the presence of *S. epidermidis* and *Enterococcus faecalis,* without fungi. The index values of serum *Aspergillus* galactomannan antigen decreased progressively, and were negative on day 30. The treatment with amphotericin B was stopped 7 days later, following the clinical improvement of the lesions, the negative fungal culture and the confirmation of the negative antigenemia on another sample. A relay by a topical treatment with econazole cream was started. A last smear of the healing abdominal skin lesions was collected on day 48 and the culture was sterile. Globally, management of some complications linked to premature birth were delayed because of the infectious context in the twin B, but the outcome was favorable. On day 66, the twin A was transferred back to the neonatal department of the general hospital where he was born (Fig. 1).

### Twin B: evolution

On admission to the NICU, the twin B received three additional doses of surfactant for hyaline membrane disease. He was treated with amoxicillin and amikacin until the CRP normalized on day 6 and with low doses of hydrocortisone during the first 10 days of life as part of the same protocol as his brother.

Six days after birth, his brother (twin A) presented abdominal cutaneous lesions leading to the removal of the umbilical catheter in both twins and their replacement by peripheral venous catheters in the axillary vein. On day 8, the culture of the umbilical catheter of twin B was also positive for *S. epidermidis* and for molds later identified as *A. fumigatus*, with a count of 20 to 40 fungal colonies on bacteriological media. Antifungal treatment with liposomal amphotericin B was started, in the hypothesis of a similar infection with *A. fumigatus* affecting the two brothers, even though twin A presented no skin lesion at this time. Incubators were replaced for both twins. The level of serum *Aspergillus* galactomannan antigen was very high on day 8, also reaching a value >5 (index). No other determination of the serum *Aspergillus* galactomannan antigen was determined for twin B. Like for his brother, *A. fumigatus* was found in low quantities in urine samples collected on day 9, but the culture of tracheal aspirations as well as blood cultures remained negative. On day 10, an erythematous yellowish cutaneous lesion appeared on the abdomen of twin B around the umbilicus. This lesion later became crusted. An empiric antibiotherapy with vancomycin was initiated with continued treatment with liposomal amphotericin B. However, the culture of a smear of these lesions remained sterile. A treatment by cefotaxime was added on day 11, replaced by imipenem on day 16 after identification of *Bacillus cereus* in a positive blood-culture.

**Fig. 2:**
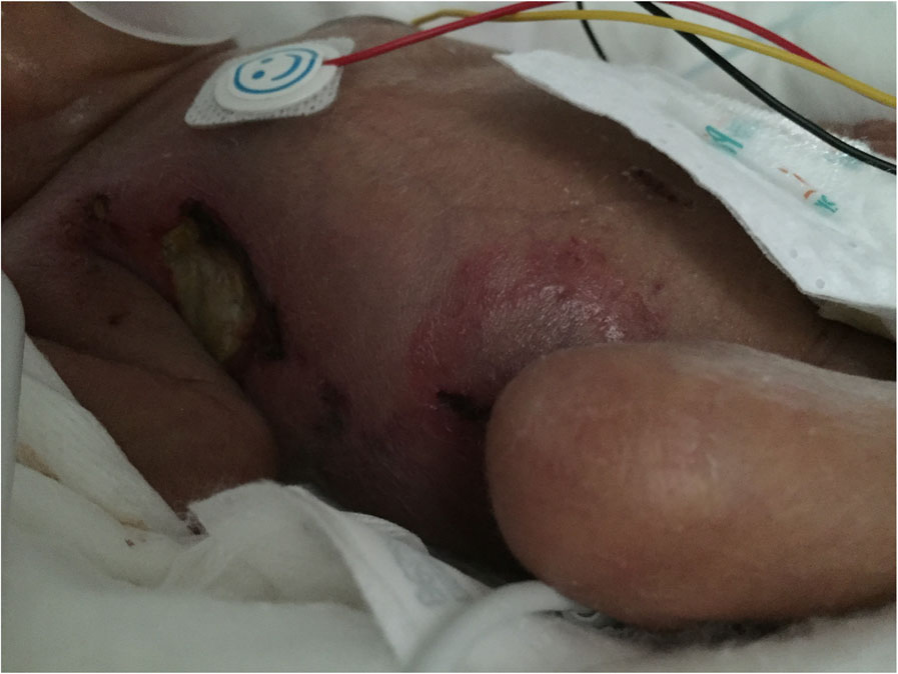
Abdominal lesions of twin B (Day 17)

Besides these infectious complications, the twin B suffered from several complications linked to premature birth. In the face of clinical deterioration with multiple organ failures despite appropriate treatments, a multidisciplinary decision in agreement with the parents was made on day 17 to limit active treatments. The twin B died the following day. No autopsy was performed (Fig. 2).

### Epidemiological investigation

**Fig. 3:**
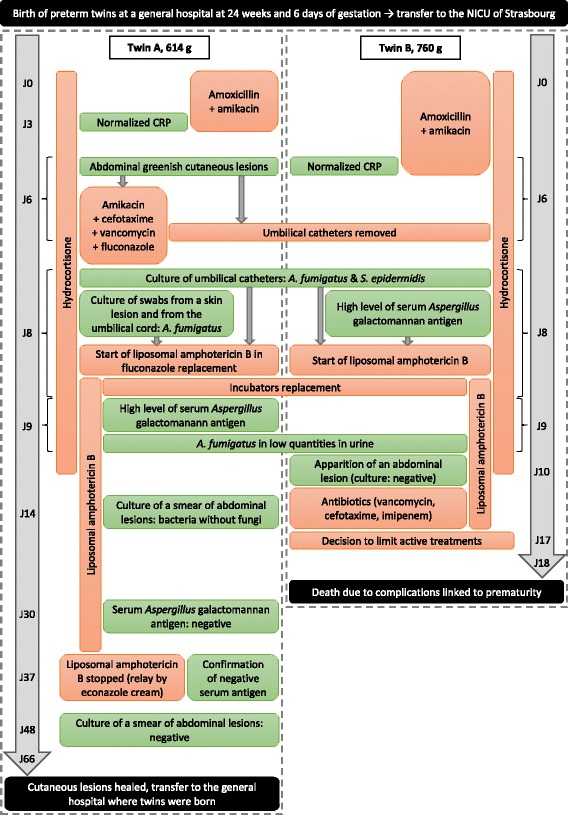
Timeline

After diagnosis of invasive aspergillosis in the twin A, an epidemiological investigation was undertaken to identify the source of contamination by *Aspergillus* and to prevent any outbreak of further cases of suspected nosocomial aspergillosis. On day 12, multiple samples had been collected in the hospital room of the twins and in the diaper storage area. Samples were also collected in the transfer incubator used the first day of life of twin B and in his incubator used until day 8, date of the diagnosis of the fungal cutaneous infection. This investigation did not enable finding the source of *A. fumigatus*. No epidemiological investigation was performed at the hospital where the twins were born. There was no construction work or renovation going on at this time in both hospitals’ surroundings, and no other cases were diagnosed in the following months (Fig. 3).

## Literature review and discussion

Including the two present cases, 41 cases of primary cutaneous aspergillosis have been published so far in reviews [3–6] and clinical cases of infection in preterm neonates (Tables 1 and 2), with a mean of gestational age of 25 weeks (23–32) and a mean of birth weight of 720 g (420–1500).

**Table 1:** Demographics, risk factors and clinical aspects of primary cutaneous aspergillosis in preterm neonates

N° [Ref]	Gestation (weeks)	Sex	Birth weight (g)	Age (days)	Antibiotics (days)	Steroids (days)	Type of skin lesions	Site of skin lesions
1 [11]	26	F	800	14	Yes (7)	NR	Single erythematous nodule with pustules	Left mid-back
2 [12]	25	M	635	5	Yes	NR	Erythematous papules with central pustules becoming necrotic ulcers	Lower back, legs
3 [19]	25	F	785	6	Yes (6)	Yes (1)	Crusted lesions with erythematous borders	Back, axillae
4 [19]	Preterm	M	670	7	Yes	No	NR	NR
5 [33]	24	M	710	6	Yes	NR	Two ulcerations surrounded by multiple abscesses	Back
6 [15]	27	M	1500	8	Yes (7)	Yes (2)	Indurated pustule surrounded by erythema becoming eroded and with marginal pustules	Foot
7 [34]	26	M	825	10	NR	NR	Maculopapular rash becoming pustular lesions	NR
8 [35]	25	NR	615	7	NR	NR	NR	NR
9 [3]	26	M	960	30	Yes	Yes (8)	Dark-red plaque and pustules	Leg
10 [13]	32	F	1470	13	Yes (7)	NR	Indurated abscess with erythematous overlying skin	Axilla
11 [16]	25	M	530	3	Yes	Yes	Greyish-white lesions becoming eschars	Thighs, penis
12 [16]	29	M	440	6	Yes	Yes	Pustules becoming eschars	Thighs, penis
13 [16]	26	M	540	5	Yes	Yes	Erythema becoming eschars	Genital area
14 [16]	26	M	715	8	Yes	No	Erythema leading to skin defects	Penis, genital area, abdomen
15 [7]	Preterm	M	900	17	Yes (17)	No	Ulcers with black eschars	Cheek, nose, ear
16 [20]	Preterm	NR	792	3	NR	NR	Necrotic skin lesions	Abdomen
17 [13]	Preterm	NR	420	7	NR	NR	Necrotic skin lesions	Abdomen
18 [21]	29	NR	1300	14	Yes	NR	Erythematous skin lesion becoming necrotic ulcers	Near the mouth
19 [5]	25	M	750	13	Yes (7)	Yes	Erythematous lesions with a central pustule and yellow crust becoming necrotic eschars	Scrotum, buttock
20 [36]	24	NR	510	12	Yes (12)	NR	Abrasion becoming necrotic eschar surrounded by erythema	Abdomen, chest, back
21 [25]	26	M	825	NR	Yes	Yes	Necrotic purpura and erythema with satellite lesions	Neck, abdomen, lower extremity
22 [25]	28	F	580	NR	Yes	No	Necrotic ulcers becoming eschar	Sacrum
23 [25]	26	M	580	NR	Yes	Yes	Crusted erosion becoming eschar	Chest tube site
24 [25]	27	F	720	NR	Yes	Yes	Necrotic purpura	Face, lower extremity
25 [25]	24	F	570	NR	Yes	No	Necrotic lesions	Back
26 [37]	24	M	653	6	Yes (6)	NR	Dark crusted efflorescences surrounded by erythema	Back
27 [32]	24	F	600	5	Yes	NR	Periumbilical smoky-grey removable patches, erythema, maceration and pustules	Umbilicus, back
28 [32]	23	M	540	7	Yes	NR	Umbilical smoky-grey removable patches, erythema and maceration	Umbilicus, leg, abdomen, chest
29 [38]	24	M	651	10	Yes (10)	NR	Moist white to yellow abrasion becoming a necrotic lesion	Back
30 [39]	27	F	750	12	Yes	NR	Erythema becoming necrotic lesions	Back
31 [17]	24	M	600	6	Yes	NR	Confluent blisters with central necrosis surrounded by erythema	Back, axillae, perineum
32 [18]	24	M	760	3	NR	NR	Purpuric and ulcerated rash becoming verrucous and crusted necrotic lesions	Back, chest, limbs
33 [18]	23	M	610	10	Yes (10)	NR	Circular papules with white eschars	Back
34 [18]	24	M	590	10	Yes (5)	NR	Circular maculopapular lesions	Back, axillae, neck
35 [40]	27	F	730	10	NR	NR	Erythematous plaque becoming hemorrhagic ulceration with abscesses	Arm, trunk, abdomen
36 [41]	25	F	490	33	NR	NR	Small hyper-pigmented nodule with a smaller satellite lesion	Hip
37 [42]	24	M	750	5–12	Yes (10d)	NR	Vesicles becoming nodules with yellow crusted central necrosis evolving into ulcers	Inguinal area
38 [43]	25	M	NR	8	Yes	NR	Single circular crusted papule with surrounding erythema	Left hip
39 [44]	25	M	550	4	Yes (4d)	NR	Erythema with elevated edges, central ulceration and white-greyish exudate	Cervical region
40 Twin A	24	M	614	6	Yes (3d)	Yes (10)	Crusted greenish ulcerations	Umbilicus, abdomen
41 Twin B	24	M	760	10	Yes (6d)	Yes (10)	Erythema becoming crusted yellowish ulcerations	Umbilicus, abdomen

*NR* not reported

**Table 2:** Diagnosis, therapeutic management and outcome of primary cutaneous aspergillosis in preterm neonates

N° [Ref]	*Aspergillus* species	Mode of diagnosis	Antifungal therapy (duration)	Surgery	Outcome
1 [11]	*A. flavus*	Stain / culture	None	Excision	Died (unrelated, age 38d)
2 [12]	*A. flavus*	Stain / culture	NR		NR
3 [19]	*A. fumigatus*	Stain / culture	AmB (60 mg/kg TD) and 5-FC (5d)		Cured/survived (FU 1 mo)
4 [19]	*A. fumigatus*	Stain / culture	None		Died (age 7d)
5 [33]	*A. fumigatus*	Stain / culture	LAmB (100,5 mg/kg TD)		Cured/survived (FU 3 mo)
6 [15]	*A. fumigatus*	Culture only	AmB (15.8 mg/kg TD)		Cured/survived (FU 40 d)
7 [34]	*A. fumigatus*	Culture only	AmB and 5-FC (4w)		Cured/survived (endophtalmitis 3 w after end of treatment)
8 [35]	*A. niger*	Stain / culture	AmB (40.5 mg/kg TD)		Cured/survived (NR)
9 [3]	*A. fumigatus*	Stain / culture	AmB (6.7 mg/kg TD)		Cured/survived (FU 1 y)
10 [13]	*Aspergillus* sp.	Culture only	AmB (32 mg TD/46d) and 5-FC (2w)	Abscess drainage	Cured/survived (age 3 mo)
11 [16]	*Aspergillus* sp.	Stain only (autopsy)	None		Died (related; age 7 d)
12 [16]	*A. fumigatus*	Culture only	None		Died (related; age 10 d)
13 [16]	*A. fumigatus, A. flavus*	Culture / serum GM (+ stain at autopsy)	5-FC (100 mg/kg/d/NR)		Died (related; time to death NR)
14 [16]	*A. fumigatus*	Culture / serum GM	LAmB (1.4–2.8 mg/kg/d/19d)		Cured/survived (age 20 mo)
15 [7]	*A. niger*	Culture only	AmB (0.25–1.0 mg/kg/d/6d)		Died (likely unrelated, time to death NR)
16 [20]	*A. flavus*	Stain / culture	NR		Died (unrelated; age 12 d)
17 [13]	*A. flavus*	Culture only	AmB 7d		Died (likely related; age 17 d)
18 [21]	*A. flavus*	Stain / culture	AmB		Died (related; time to death NR)
19 [5]	*A. fumigatus*	Stain / culture	AmB (1.5 mg/kg/d/22d)		Died (unrelated; age 40 d)
20 [36]	*A. flavus*	Stain / culture	LAmB (5 mg/kg/d /NR, after empiric AmB and fluconazole)		Died (likely related, time to death NR)
21 [25]	*A. fumigatus, A. flavus*	Stain / culture	AmB		Died (time to death NR)
22 [25]	*A. flavus*	Stain / culture	AmB		Died (time to death NR)
23 [25]	*A. flavus*	Stain / culture	AmB and 5-FC		Cured/survived (age NR)
24 [25]	*A. flavus*	Stain / culture	AmB		Died (time to death NR)
25 [25]	*A. fumigatus*	Stain / culture	AmB		Cured/survived (age NR)
26 [37]	*A. fumigatus*	Culture only	AmB (32 mg/kg TD, 40 d)		Cured/survived (age 146 d)
27 [32]	*A. fumigatus*	Culture only	LAmB (28d), then voriconazole (55d). Topical gentian violet and cyclopiroxolamine		Cured/survived (age NR)
28 [25]	*A. niger*	Culture only	LAmB (5d), then voriconazole (28d). Topical gentian violet and cyclopiroxolamine		Cured/survived (age NR)
29 [38]	*A. fumigatus*	Stain/culture	AmB lipid complex (7 mg/kg/d/3d), then LAmB (5 mg/kg/d/3w) + voriconazole (8 mg/kg/d/7w) + caspofungin (2 mg/kg/d/1w) replaced by micafungin (8 mg/kg/d/6w)		Cured/survived (19 mo)
30 [39]	*A. fumigatus*	Stain/culture	LAmB (5 mg/kg/d/10d)		Died (unrelated; age 16 d)
31 [17]	*A. fumigatus*	Culture (+ stain at autopsy)	LAmB (3,5 mg/kg/d/2d)		Died (related; age 13 d)
32 [18]	*A. fumigatus*	Stain / culture	AmB lipid complex (1d)		Died (likely related; age 6 d)
33 [18]	*A. fumigatus*	Stain / culture	AmB lipid complex alone then + caspofungin (total: 22d), then posaconazole (29d)		Cured/survived (age NR)
34 [18]	*A. fumigatus*	Stain / culture	AmB lipid complex (15d), then oral posaconazole (21d)		Cured/survived (age NR)
35 [40]	*A. flavus*	Stain / culture	LAmB (3 mg/kg/d/28d), then itraconazole (1 mg/kg/d/30d)		Cured/survived (age 3 y)
36 [41]	*A. fumigatus*	Stain / culture	LAmB (3 mg/kg/d/8d)	Excision	Cured/survived (age 3 y)
37 [42]	*A. fumigatus*	Stain / culture	LAmB (5 mg/kg/d/14d)	Excision	Cured/survived (age 20 mo)
38 [43]	*A. fumigatus*	Stain / culture	LAmB (21d)		Cured/survived (age NR)
39 [44]	*A. fumigatus, A. nidulans*	Culture / serum GM	LAmB (5 mg/kg/d/11d) alone then + voriconazole (6 mg/kg/d/2d, stopped because of toxic serum concentrations)		Died (unrelated; age 22 d)
40 Twin A	*A. fumigatus*	Culture / serum GM	LAmB (5 mg/kg/d/29d)		Cured/survived (age 66 d)
41 Twin B	*A. fumigatus*	Culture / serum GM	LAmB (5 mg/kg/d/9d)		Died (likely unrelated, age 18 d)

*NR* not reported, *GM* galactomannan, *AmB* Amphotericin B deoxycholate, *LAmB* liposomal Amphotericin B, *5-FC* 5-fluorocytosine, *TD* Total dose; *FU* Time to follow-up after end of treatment

Preterm infants have an important predisposition to neonatal PCA [4, 7]. This may be the result of a functionally immature immune system and immature skin barrier [1]. Indeed, their skin presents an increased vulnerability to minor trauma associated with intensive care. The skin of newborns is so fragile that a minor friction can allow *Aspergillus* spores to enter through a breach in the epithelium [1]. Another PCA risk factor is neutropenia, with an infrequent occurrence in neonates with aspergillosis [4]. This population may rather have a predisposition to aspergillosis through a qualitative defect in macrophages and neutrophil chemotaxis, phagocytosis and microbicidal activity, especially under severe illness and stress [1]. Other known risk factors include glucocorticoid administration and prior use of antibiotics [4, 5]. Macrophages normally ingest and kill *Aspergillus* spores and prevent germination, whereas normal neutrophil granulocytes stop hyphal growth and dissemination, and eradicate mycelia [8]. Glucocorticoids have been shown to impair the number as well as the fungicidal activity of both neutrophils and macrophages against *Aspergillus* [8–10]. Here, both twins received hydrocortisone during their first 10 days of life in order to prevent bronchopulmonary dysplasia. The exact role of antibiotics in the pathogenesis of PCA is unclear, but they may contribute to the infection by disturbing the skin flora ecology [11–14]. In the present report, *Aspergillus* infection first appeared 6 days after the birth of twin A, so after only a short cure of antibiotics. Prior exposure to antibiotics was documented in 34 reported cases of PCA (83%) and prior exposure to glucocorticoid in 12 cases (29%).

The mean age of neonates when first lesions appeared was 10 days, ranging from 3 to 33 days. Clinical presentations of PCA are not specific (Table 1). Typically, PCA begins as erythematous papule(s) or plaque(s). The skin lesions progress to pustules and to yellow crusted ulcerations like in our cases, and eventually to necrotic black eschars. An atypical form was described in the literature as a single indurated abscess with erythematous overlying skin [13]. Thus, any new skin lesion in a neonate at risk should raise the suspicion of PCA.

*A. fumigatus* is the species most frequently encountered in this type of infection (26 cases, 63%), followed by *A. flavus* (12 cases, 29%) and *A. niger* (3 cases, 7%). In 2 reported cases, 2 different species of *Aspergillus* sp. have been identified. The diagnosis was limited to *Aspergillus* sp. in one other case, one being only “suggested” after tissue staining. Except for the latter case, all infections were diagnosed at least with a positive culture. There was positive *Aspergillus* galactomannan antigenemia in 5 cases (12%) and positive tissue staining in 28 cases (68%), only after autopsy in 3 cases.

In most reported cases of neonatal PCA, the source of contamination was not discovered [4]. Construction in hospital areas close to immunocompromised patients is a well-described risk factor for *Aspergillus* infections. Construction work at or near the hospital was documented in 12 reported cases (29%). Other presumed or documented sources include oximeter sensors [15], latex finger stalls [16], non-sterile gloves [17], humidifying chambers of incubators [18], bedding [19] and adhesive tapes [3, 7, 11, 20, 21]. These sources of *Aspergillus* are also causes of maceration that can lead to small breaches in the skin, allowing *Aspergillus* spores to invade [1]. No hypothesis was made about the source of infection or the portal of entry in 21 of the reported cases (51%).

In the present report, the infection appeared shortly after birth and approximatively at the same time in both twins, suggesting a common source of contamination soon after birth, or maybe even during delivery. The umbilical localization is also quite uncommon and raises the question of a potential mother-to-child transmission. *Aspergillus* species are ubiquitous in nature and have been isolated from the human vaginal tract [22]. In cattle, they may infect the placenta and cause abortion [23]. In a report from the former U.R.S.S., human intra-uterine infection has been suggested on the basis of histopathologic findings in 6 stillborn fetuses [24]. However, a real proof for this route of infection could not be provided. Although contact transmission can occur, airborne transmission of *Aspergillus* spores is the major route of infection. In this case, no samples from the mother were sent to the microbiology laboratory before or after delivery.

The father of twins worked in the waste management sector and had regular skin-to-skin contact with twins after their birth, which could have been a potential source of contamination; this hypothesis was not explored further. The epidemiological investigation did not enable finding the source of contamination and no construction work was in progress at that time. Central umbilical catheters were positive for *A. fumigatus* in both twins before the occurrence of the cutaneous lesions in twin B, suggesting that theses catheters played a role in the infection. Skin maceration was another risk factor since the lesions appeared on the abdomen under the diapers. Besides the catheters and the diapers, contaminated materials used for both siblings or cross-contamination by medical personnel are the most likely sources. The contamination might have occurred at the outside hospital, during transport, or in the ICU.

Primary cutaneous aspergillosis can be a site-specific entity or the initial point of invasive or disseminated disease. In the literature, autopsy findings enabled to conclude to a disseminated aspergillosis in 6 of the 10 cases for which an autopsy was performed [13, 16–18, 21, 25]. In the present cases, no autopsy was performed in the case of twin B. Levels of serum *Aspergillus* galactomannan antigen were very high in both twins on days 8–9 and they remained positive for 3 weeks in the case of twin A. False positive serum *Aspergillus* galactomannan levels have been described outside of an invasive aspergillosis context in cases of intestinal fungal colonization, cross-reactivity with unidentified serum compounds or in cases of bacteremia or fungemia [26–28]. No stool samples from our two patients had been collected and there were no bacteremia or fungemia episodes concomitant with the positivity of the galactomannan. Furthermore, twins A and B had received 3 and 6 days of amoxicillin right after birth respectively, an antibiotic linked to false positive serum *Aspergillus* galactomannan levels [28, 29]. However in the case of twin A, the serum *Aspergillus* galactomannan levels were found positive on day 9, meaning 6 days after interruption of the amoxicillin treatment, and the negativation of this marker was in concordance with his clinical evolution. These data together with the ulcerative aspect of the skin lesions are arguments in favor of an invasive aspergillosis in both twins. In addition, *A. fumigatus* was found in low quantity in the urine of both twins at the same time as the cutaneous infection and this could be an element in favor of a disseminated infection. However, a contamination of urine samples by *Aspergillus* via compresses in the diapers can’t be excluded.

Most of the reported cases of PCA have been treated primarily with systemic deoxycholate amphotericin B. It remains the first-line therapy with a dose of 1 mg/kg/day for both suspected and proven primary invasive cutaneous aspergillosis in neonates, sometimes in combination with 5-fluorocytosine [4, 30, 31]. The experience with liposomal amphotericin B (Ambisome^®^) as a treatment of PCA is still limited in neonates of low birth weight. However, liposomal amphotericin B was the treatment given in the last reported cases of PCA. The treatment used in the present report is one of the alternative therapies with a dosage of 5 mg/kg/day [4, 30]. In cases of PCA refractory to amphotericin B, successful treatment with voriconazole has been reported in the literature [32]. Surgical debridement may also improve survival [30]. However, surgery is often not possible because neonates tend not to tolerate skin surgery well, and lesions can be extensive [1]. Surgical intervention was performed only in four of reported cases (10%). Prompt diagnosis and treatment maximize the chances of a favorable outcome. Nineteen neonates (46%) died after PCA in reported cases, with the death related or likely related to aspergillosis in eight cases (42% of dead patients). In the present report, invasive PCA was rapidly diagnosed and an adapted treatment was started promptly, but one of the two brothers died because of several other complications linked to premature birth.

## Conclusion

The skin fragility of preterm newborns is an excellent potential entry point for environmental fungal infections. These cases highlight the importance of suspecting PCA in extremely low birth weight neonates with rapidly progressive necrotic lesions. An empiric antifungal treatment should be started promptly at the first signs of cutaneous infection, just after samples collection. In case of twins, the brother should be very closely monitored.

## Abbreviations

5-FC: 5-fluorocytosine
AmB: Amphotericin B deoxycholate
CRP: C reactive protein
FU: Time to follow-up after end of treatment
GM: Galactomannan
LAmB: liposomal amphotericin B
NICU: Neonatal Intensive Care Unit
NR: Not reported
PCA: Primary cutaneous aspergillosis
TD: Total dose
